# Upregulation of microRNA‐762 suppresses the expression of GIPC3 in systemic lupus erythematosus and neuropsychiatric systemic lupus erythematosus

**DOI:** 10.1002/iid3.719

**Published:** 2022-10-11

**Authors:** Jijuan Yang, Chun Li, Shuhong Chi, Hongliang Wei, Wenqing Du, Qikuan Hu

**Affiliations:** ^1^ Department of Rheumatology General Hospital of Ningxia Medical University Yinchuan Ningxia P.R.China; ^2^ Department of Nursing Xingtai Medical College Xingtai Hebei P.R.China; ^3^ Department of Emergency The First People's Hospital of Yinchuan Yinchuan Ningxia P.R.China; ^4^ Department of Physiology Ningxia Medical University Yinchuan Ningxia P.R.China; ^5^ Ningxia Key Laboratory of Cerebrocranial Disease, Basic Medical School of Ningxia Medical University Yinchuan Ningxia P.R.China

**Keywords:** GIPC3, microRNA‐762, neuropsychiatric systemic lupus erythematosus (NPSLE), systemic lupus erythematosus (SLE)

## Abstract

**Background:**

Systemic lupus erythematosus (SLE), especially neuropsychiatric SLE (NPSLE), is a complex systemic autoimmune disease, characterized by variable course and multiple organ dysfunction. Our study aimed to identify crucial microRNA (miRNAs) in SLE and NPSLE.

**Methods:**

Totally 12 cases of serum specimens were collected from General Hospital of Ningxia Medical University (SLE = 4, NPSLE = 4, control = 4). After miRNA sequencing, differential expression analysis, miRNA target prediction, and miRNA‐messenger RNA (mRNA) regulatory network construction were performed to identify the hub miRNAs. The expression of target gene was determined by quantitative reverse transcription‐polymerase chain reaction and Western blot.

**Results:**

There were 79 and 59 differentially expressed miRNAs (DEmiRNAs) in NPSLE versus Control, and SLE versus Control, respectively. Among 35 overlapped DEmiRNAs, 5 upregulated miRNAs' (hsa‐miR‐762, hsa‐miR‐4270, hsa‐miR‐3663‐3p, hsa‐miR‐4778‐5p, and hsa‐miR‐4516) target genes were supported by at least six databases. The miRNA‐mRNA network indicated that core miRNA hsa‐miR‐762 regulated 1270 target genes. MiR‐762 was significantly upregulated in SLE and NPSLE, and over expression of miR‐762 significantly suppressed GIPC PDZ domain containing family member 3 (GIPC3) expression in SLE and NPSLE.

**Conclusions:**

Upregulation of hub miRNA miR‐762 can suppress the expression of GIPC3 in both SLE and NPSLE samples, which is probably involved in the development of SLE and NPSLE. Meanwhile, along with the development from SLE to NPSLE, miR‐762 exhibits higher expression.

## Graphical abstract

In this study, we found that miR‐762 was significantly upregulated in Systemic lupus erythematosus (SLE) and neuropsychiatric SLE (NPSLE), and overexpression of miR‐762 significantly suppressed GIPC3 expression. MiR‐762 potentially contributes to the development of SLE and NPSLE, besides higher hsa‐miR‐762 expression might promote the development from SLE to NPSLE via inhibiting GIPC3 expression.

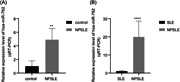

## 1 INTRODUCTION

Systemic lupus erythematosus (SLE) is a complex chronic, inflammatory systemic autoimmune disease involving innate and adaptive immune responses, usually characterized by flare, variable course, and multiple organ dysfunction.^1^, ^2^, ^3^ Although the underling molecular mechanisms of SLE are still not fully clarified, the autologous nucleic acids are attacked by immune responses in SLE patients resulting from multiple genetic and environmental factors.^4^, ^5^ Meanwhile, the accumulation of overproduced autoantibodies would lead to the injury of various organs, such as kidney, skin, heart, and lungs.^6^ Accordingly, the mortality rate of SLE patients is almost three times higher than healthy individuals, and the rate will increase as the disease progression.^7^ Neuropsychiatric systemic lupus erythematosus (NPSLE) is a type of severe complication of SLE.^8^ NPSLE implicates not only central nervous systems but also peripheral nervous systems, and it is one of the main cause of mortality in SLE patients, only surpassed by lupus nephritis.^9^, ^10^ The manifestations of NPSLE are usually heterogeneous, indicating from localized or to diffuse, or from mild to severe.^11^ Owing to lacking of specific and sensitive methods, the diagnosis of NPSLE is still a great challenge for rheumatologists.^8^ Currently, two aspects of pathologic changes are usually observed in NPSLE patients, including autoimmune/inflammatory pathways alterations caused dysfunctional blood‐brain barrier or intrathecal immune complex accumulation, and ischemic or thrombotic pathway caused vascular occlusion events.^12^, ^13^ Undoubtedly, deepening understanding of the mechanisms of SLE and NPSLE development would be conducive to better management strategies for patients.

Growing evidence has recently highlighted the potential role of noncoding RNAs, especially microRNAs (miRNAs), in innate and adaptive immune responses in SLE and NPSLE.^14^ For instance, Garchow et al. have emphasized the role of aberrant gene and miRNA expression in SLE, suggesting that elevated miR‐210 expression is observed in CD4+ cells from lupus patients and lupus‐prone mouse models.^15^ Mishra et al. have documented the regulatory role of miR‐30e in innate immunity in SLE, involving the regulation of type‐I interferon and proinflammatory cytokines.^16^ In vivo evidence indicates that long noncoding RNAs myocardial infarction associated transcript enhances the activity of SLE by upregulating miR‐222 and CFHR5 expression.^17^ The expression of miR‐145, miR‐223 and miR‐326 in NPSLE patients were significantly downregulated, which has been reported as possible diagnostic biomarkers.^18^ The above information indicates the great potential of miRNAs in SLE and NPSLE. However, as far as we know, few reports have focused on the role of miRNA in SLE and NPSLE meanwhile, especially hsa‐miR‐762.

Herein, based on our local clinical SLE and NPSLE samples, we expect to explore the potential role of hub miRNAs using miRNA sequencing and bioinformatics tools. Our findings would contribute to better understanding of possible mechanisms in SLE and NPSLE, and might be helpful for the diagnosis of SLE and NPSLE.

## 2 MATERIALS AND METHODS

### 2.1 Sample collection

In present study, we totally collected 12 cases of human serum specimens from General Hospital of Ningxia Medical University, comprising 4 NPSLE samples, 4 SLE samples, and 4 normal control samples. The NPSLE samples were collected from August 2016 to October 2018, and SLE and control samples were collected during March 2018−September 2018. The experiments were approved by ethics committee of hospital, in line with The Helsinki Declaration. Informed consents were obtained from all donors, and the detailed clinical information was displayed in Supporting Informaton: Table S1.

The human miRNA expression profile of all samples were detected using Agilent Human miRNA Microarray Kit Release 21.0, 8x60K chip.

### 2.2 Inclusion and exclusion criteria

All NPSLE participants accorded with American College of Rheumatology Criteria for SLE (1999). Meanwhile, the following patients were excluded: (1) Patients with neuropsychiatric symptoms before the diagnosis of SLE/NPSLE; (2) NPSLE patients caused by traumatic brain trauma, intracranial infection, hypoxemia, hepatic encephalopathy, uremia, tumors, or severe electrolyte imbalance; (3) Patients with genetic risk factors.

The SLE patients were in accordance with American College of Rheumatology Criteria for SLE (1997), meanwhile there was no neuropsychiatric symptom.

Normal participants all had free history of chronic diseases.

### 2.3 Differentially expressed miRNAs (DEmiRNAs)

The tiff format picture data after chip scanning was preprocessed utilizing Feature Extraction software, and the raw data files (.txt) were obtained. Then, the raw data was input in GeneSpring software, and parameter information such as group information was written. All samples' data was normalized and undergone quality control. DEmiRNAs were analyzed, and *p* value was calculated.

### 2.4 Target gene prediction of miRNA

Regarding the DEmiRNAs, we have predicted their target genes in 12 software, including miRWalk, DIANA‐microTv4.0, miRanda‐rel2010, mirBridge, miRDB4.0, miRmap, miRNAMap, PicTar2, PITA, RNA22v2, RNAhybrid2.1, and Targetscan6.2. Target genes predicted in at least six software programs in the meantime were considered to be DEmiRNAs' final targets.

### 2.5 Functional enrichment analysis

Additionally, the target genes were subjected to the functional enrichment analysis in clusterProfiler package of R,^19^ comprising gene ontology (GO) and Kyoto Encyclopedia of Genes and Genomes (KEGG) enrichment analysis. For screening significantly enriched terms, the threshold was set at *p* adjust <.05.

### 2.6 Cell lines

HT22 mouse hippocampal neuronal cell line was purchased from Jennio Biological Technology. The cells were cultured in high‐glucose Dulbecco's modified Eagle's medium (SH30022.01B; Hyclone), supplemented with 1% penicillin and 10% fetal bovine serum (FB15015, CLARK), in a 37°C and 5% CO_2_ incubator.

### 2.7 Quantitative reverse transcription‐polymerase chain reaction assay

Serum miRNA was extracted using miRNeasy Mini Kit (217004; QIAGEN). After RNA extraction, agarose gel electrophoresis was used to detect the RNA integrity. The concentration and purity of RNA was determined by UV spectrophotometer, and 1.9 < OD260/280 < 2.1 and OD260/230 > 2.0 indicated qualified RNA purity and concentration, respectively. The miScript SYBR Green PCR Kit (218073; QIAGEN) and miScript II RT kit (218160; QIAGEN) were used for reverse transcription and qPCR, separately. External reference was cel‐miR‐39. The procedure was as below: 95°C for 15 min, 94°C for 15 s, 55°C for 30 s, 70°C for 30 s, 40 cycles.

The miRNAs in cells were extracted with miRcute miRNA isolation kit (DP501; TIANGEN). The reagents used in qPCR included miRcute Plus miRNA qPCR Kit (FP411; TIANGEN) and FastKing RT Kit (KR116; TIANGEN). Internal reference was U6. Following procedure was conducted: 95°C for 15 min, 40 cycles of 94°C for 20 s and 60°C for 34 s.

Total RNA in cells was extracted using RNAsimple Total RNA kit (DP419; TIANGEN). TIANScript RT kit (KR107; TIANGEN) and SuperReal PreMix Plus (SYBR‐Green) kit (FP205; TIANGEN) were utilized in qPCR assay, and qPCR was done on Bio‐Rad CFX Manager 3.0. Internal reference was GAPDH. All primer sequences were designed using software Primer5 and were shown in Table 1 (three repeats per sample). The program was performed as below: 95°C for 15 min, 95°C for 10 s, 60°C for 30 s, 40 cycles. The relative mRNA expression levels were calculated according to the $2^{–\Delta \Delta C_{T}}$ formula.

**Table 1:** Primer sequences for RT‐PCR

Genes	Forward primer (5'−3')	Reverse primer (5'−3')	Product(bp) length (bp)
hsa‐miR‐762	GGGGCTGGGGCCGGGGC	‐	‐
U6	CTCGCTTCGGCAGCACA	‐	‐
GIPC3	AACGGAGGATGCTCTAGGACTGAC	ATCCGGTTGATAATGCTGCCTTCC	93
GAPDH	CAGGAGGCATTGCTGATGAT	GAAGGCTGGGGCTCATTT	138

Abbreviation: GIPC3: GIPC PDZ domain containing family member 3:  RT‐PCR, reverse transcription‐polymerase chain reaction.

### 2.8 Western blot assay

Total protein was extracted from all samples, and the concentration was determined with bicinchoninic acid protein concentration assay kit (p0012; Beyotime). The Western blot assay was done in the previous methods.^20^ The internal reference was β‐tubulin (ab21058; Abcam). The primary antibody GIPC PDZ domain containing family member 3 (GIPC3) antibody (sc‐517166; 1:200; Santa Cruz Biotechnology), secondary antibody of β‐tubulin goat anti‐rabbit (925‐68071; 1:5000, IRDye® 680RD), and secondary antibody of GIPC3 goat anti‐mouse (925‐68070; 1:6000, IRDye® 680RD) were used in our study. Densitometric analysis was finished in ImageJ software.

## 3 RESULTS

### 3.1 DEmiRNAs in SLE and NPSLE

**Figure 1:**
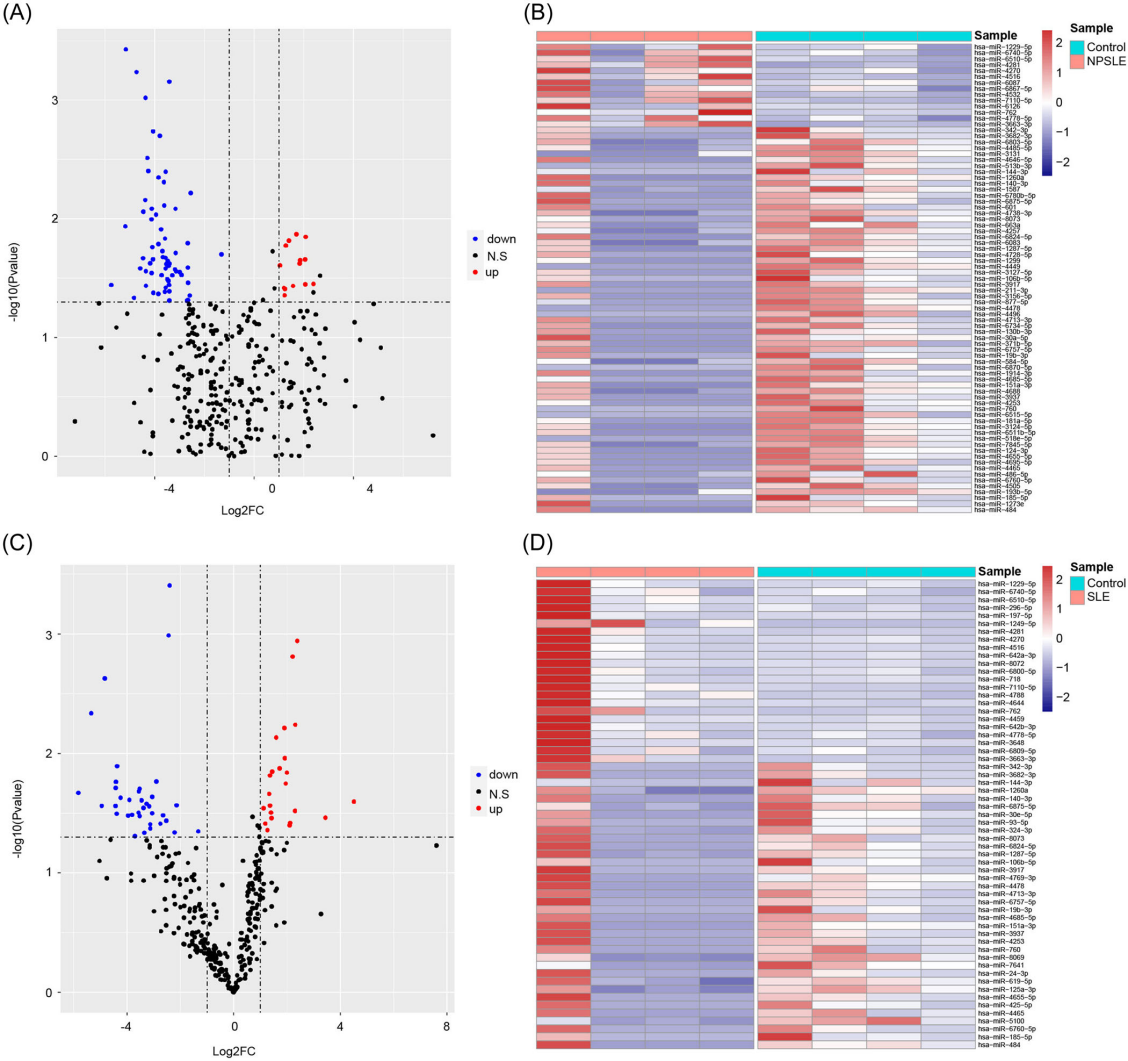
Differentially expressed miRNAs. (A, B) The volcano and heat map of DEmiRNAs between NPSLE versus Control, respectively. In heat map, blue: low expression; red: high expression. (C, D) The volcano and heat map of DEmiRNAs between SLE versus Control, respectively. In heat map, blue: low expression; red: high expression. miRNAs, microRNAs; NPSLE, neuropsychiatric systemic lupus erythematosus; SLE, ystemic lupus erythematosus.

Firstly, we have identified the DEmiRNAs in NPSLE versus Control, SLE versus Control, respectively. Compared with normal samples, there were totally 79 DEmiRNAs in NPSLE specimens, including 14 upregulated miRNAs and 65 downregulated miRNAs (Figure 1A). Expression levels of these DEmiRNAs were significantly different between NPSLE and control samples (Figure 1B). The genome distribution of some DEmiRNAs was shown in Supporting Information: Figure S1A.

Moreover, compare with normal samples, a total of 59 DEmiRNAs were identified in SLE specimens, comprising 23 upregulated miRNAs and 36 downregulated miRNAs (Figure 1C), and DEmiRNAs' expression levels were significantly different (Figure 1D). Some DEmiRNAs' distribution on genome was displayed in Supporting Informaton: Figure S1B.

### 3.2 Results of functional enrichment analysis

**Figure 2:**
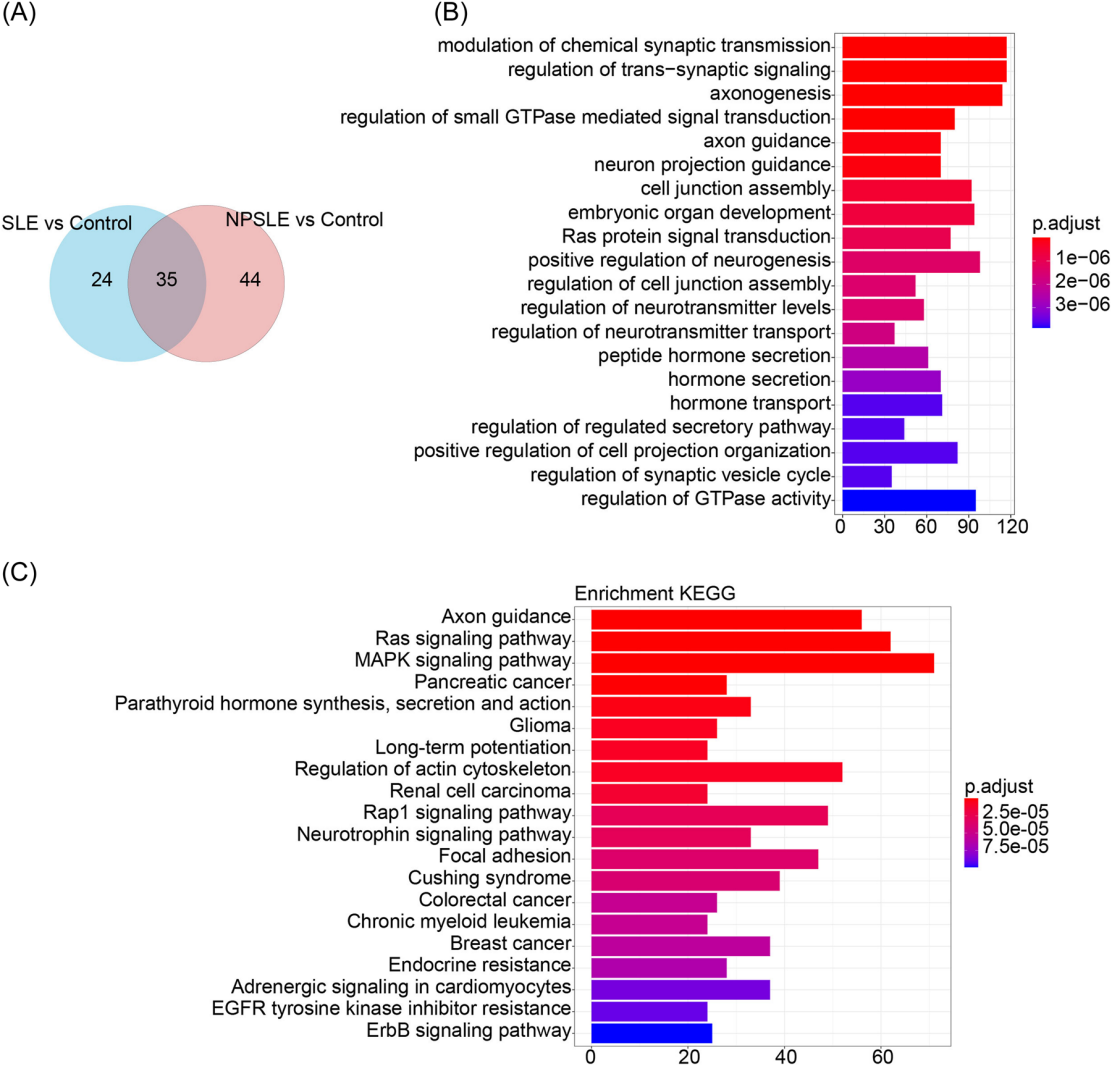
Results of functional enrichment analysis. (A) Venn diagram of DEmiRNAs in NPSLE versus Control and SLE versus Control. (B, C) The top 20 significantly enriched GO terms and KEGG pathways, separately. X‐axis: the number of genes; Y‐axis: titles of GO/KEGG terms. GO, gene ontology; KEGG, Kyoto Encyclopedia of Genes and Genomes; miRNAs, microRNAs; NPSLE, neuropsychiatric systemic lupus erythematosus; SLE, ystemic lupus erythematosus.

There were 79 and 59 DEmiRNAs in NPSLE versus Control and SLE versus Control, separately. After cross analysis of these DEmiRNAs, 35 overlapped miRNAs were obtained (Figure 2A). Among them, we found that 10 miRNAs were both upregulated in NPSLE and SLE groups. We then conducted target gene prediction on these 10 miRNAs. Our results indicated that the target genes of 5 miRNAs, hsa‐miR‐762, hsa‐miR‐4270, hsa‐miR‐3663‐3p, hsa‐miR‐4778‐5p and hsa ‐miR‐4516, were supported by at least six databases meanwhile.

There were totally 2319 non‐repetitive target genes of the above 5 miRNAs, which were subjected to GO and KEGG enrichment analysis. Those 2319 genes were significantly enriched in a total of 787 GO terms (*p* value. adjust <.05), including 623 Biological Process terms, 63 Molecular Function terms, and 101 Cellular Component terms, and the top 20 GO terms were displayed in Figure 2B. Besides, these genes were significantly enriched in 112 KEGG pathways, the top 20 of which were shown in Figure 2C. All detailed results of functional enrichment analysis were listed in Supporting Information: Table S2.

### 3.3 Construction of miRNA‐mRNA regulatory network

Using cytoscape software, a regulatory network basing on 5 miRNAs and the corresponding 2319 target genes was constructed. Our results suggested that hsa‐miR‐762 regulated the most target genes, achieving 1270 (Supporting Information: Figure S2).

### 3.4 Significantly higher hsa‐miR‐762 expression was observed in clinical NPSLE samples

**Figure 3:**
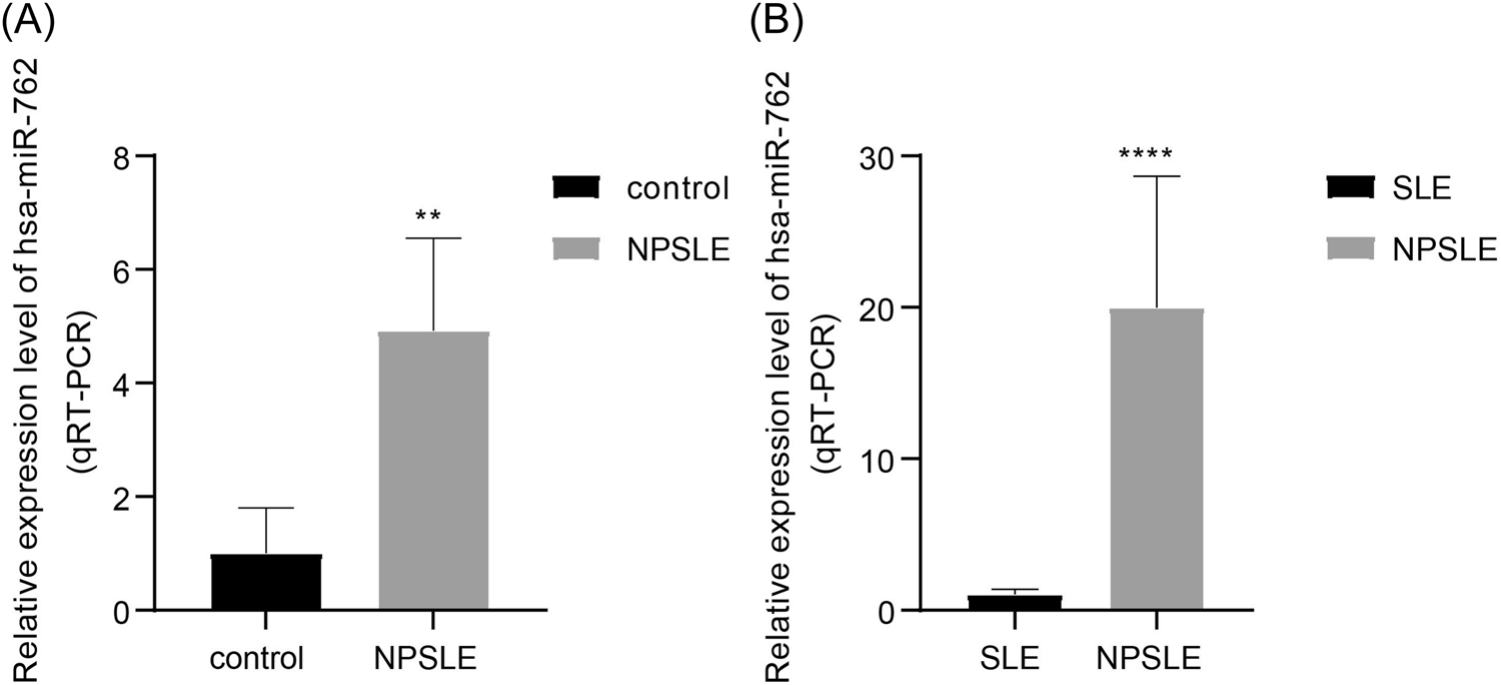
The hsa‐miR‐762 expression in clinical samples. (A, B) Compared with controls (*n* = 4) and SLE samples (*n* = 4), hsa‐miR‐762 showed significantly higher expression in NPSLE samples (*n* = 4). *p < .05, **p < .01, ***p < .001. NPSLE, neuropsychiatric systemic lupus erythematosus; SLE, ystemic lupus erythematosus.

Subsequently, we mainly focused on the differentially expressed hsa‐miR‐762 in our local clinical samples. Compared with control samples, hsa‐miR‐762 expression level was significantly higher in NPSLE samples (Figure 3A). Moreover, hsa‐miR‐762 also showed significantly higher expression level in NPSLE samples compared with SLE samples (Figure 3B). Accordingly, compared with control samples, hsa‐miR‐762 exhibited differentially higher expression levels in SLE and NPSLE samples. Our experimental results showed same tendency with the results of bioinformatics analysis, which further indicated that significantly high expression of hsa‐miR‐762 was closely correlated with the onset of NPSLE.

### 3.5 Hsa‐miR‐762 negatively regulated the GIPC3 expression

**Figure 4:**
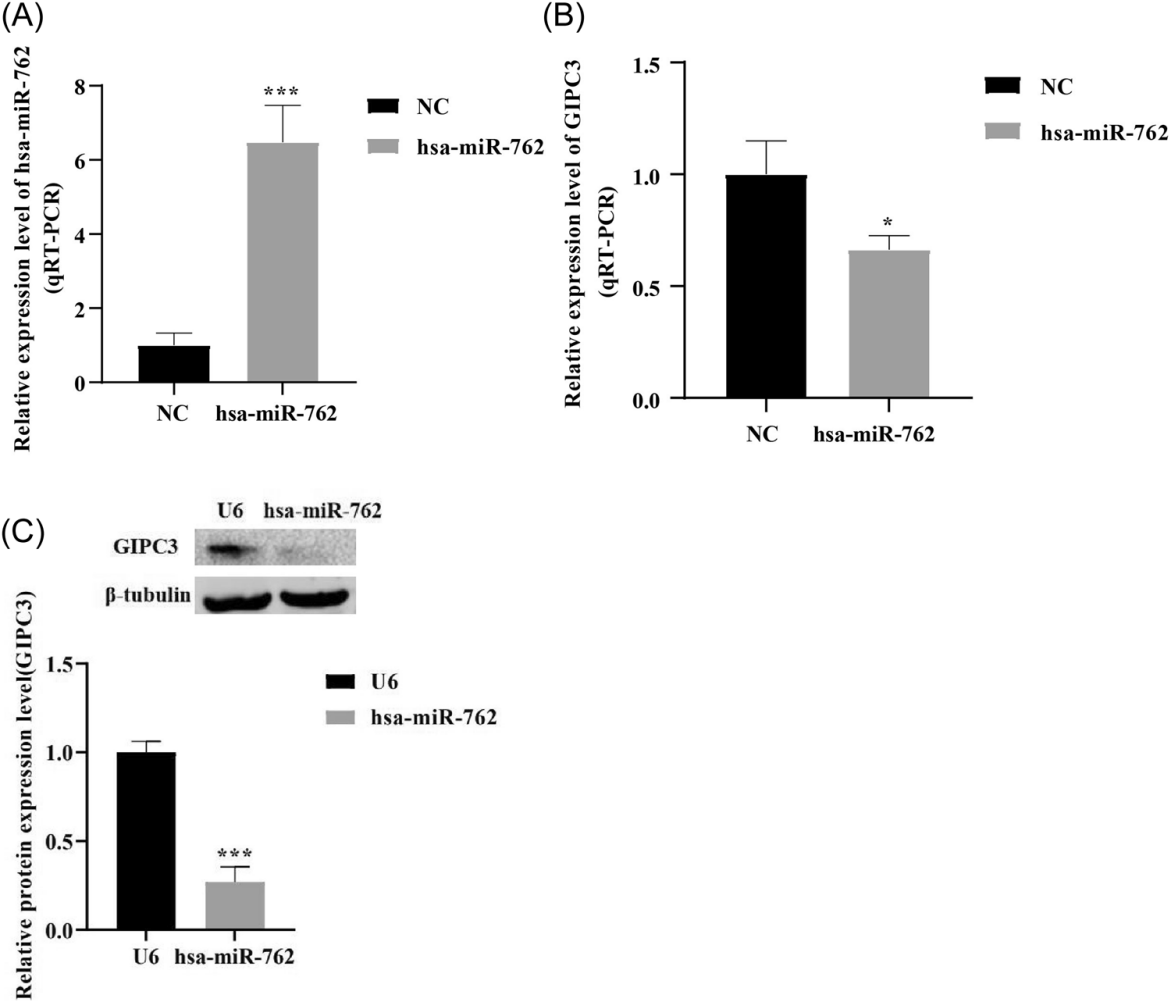
Hsa‐miR‐762 negatively regulated the GIPC3 expression in hsa‐miR‐762 overexpressed HT22 cells. (A) Over expression vector of hsa‐miR‐762 in HT22 cells was successfully constructed. (B, C) GIPC3 mRNA expression and protein expression levels in hsa‐miR‐762 overexpressed HT22 cells, respectively. *p < .05, **p < .01, ***p < .001, ****p < .0001. GIPC3, GIPC PDZ domain containing family member 3; mRNA, messenger RNA.

Basing on the prediction of Targetscan 6.2 database, GIPC3 had the highest score among all target genes of hsa‐miR‐762. Therefore, the potential influence of hsa‐miR‐762 on GIPC3 was explored. First, the over expression vector of hsa‐miR‐762 in HT22 cells was successfully constructed, the qPCR results indicated the hsa‐miR‐762 was markedly overexpressed in HT22 cells (Figure 4A). Then, we found that compared with control samples, GIPC3 mRNA expression and protein expression levels were both significantly downregulated in hsa‐miR‐762 overexpressed HT22 cells (Figure 4B−C). Our data implied that hsa‐miR‐762 potentially negatively regulated the GIPC3 expression in NPSLE samples.

## 4 DISCUSSION

SLE has been a great threat to the health, especially in females.^1^ Among which, the management of NPSLE patients is extremely challenging due to heterogeneous clinical phenotypes.^21^ Therefore, it is imperative to explore the potential molecular mechanisms and pathogenic factors in SLE and NPSLE, to provide more reference information for clinical treatments. In present study, the miRNA sequencing data of healthy controls, SLE and NPSLE samples were compared and analyzed, we found that hsa‐miR‐762 was probably a pathogenic factor in SLE and NPSLE.

First, we have identified 79 and 59 DEmiRNAs in NPSLE versus Control, SLE versus Control, respectively. After cross analysis, a total of 35 overlapped DEmiRNAs were obtained, which were more important miRNAs in the development of SLE and NPSLE. We believed that those miRNAs upregulated both in SLE and NPSLE samples were probably pathogenic miRNAs of SLE and NPSLE, which were more crucial in the development of SLE and NPSLE. Totally 10 upregulated miRNAs were identified. Among which, 5 miRNAs (hsa‐miR‐762, hsa‐miR‐4270, hsa‐miR‐3663‐3p, hsa‐miR‐4778‐5p, and hsa‐miR‐4516) with more robust target genes were subjected to the subsequent analysis. The miRNA‐mRNA regulatory network indicated that hsa‐miR‐762 was the core miRNA regulating more than half target genes. Moreover, hsa‐miR‐762 showed significantly higher expression in clinical NPSLE samples, compared with SLE or control samples. To the best of our knowledge, miR‐762 has been seldom reported in SLE or NPSLE at present. However, the role of miR‐762 has been explored in various cancers. In nasopharyngeal carcinoma, upregulation of miR‐762 was suggested to associate with progression by promoting tumor cells' proliferation and invasion.^22^ The miR‐762 has been reported to involve in the circRNA‐miRNA‐mRNA network in lung cancer as diagnostic or prognostic factor.^23^, ^24^ Although the underlying links between SLE/NPSLE and various cancers remain unclear, it's no doubt that immune dysfunction is the common ground of these diseases.^25^ Additionally, latest research of Gaines et al. demonstrated that in human astrocytes, miR‐762 was significantly associated with the genes involving neurological dysfunction.^26^ Interestingly, a recent study has revealed that upregulated miR‐762 was observed in Graves' disease patients when compared with healthy controls.^27^ Their work firstly reported miR‐762 in autoimmune disease, which could be combined with our results implying its possible role in autoimmune.

Subsequently, we mainly focused on miR‐762 and a key target gene GIPC3. Our data suggested that hsa‐miR‐762 expression was significantly upregulated in SLE and NPSLE, compared with control samples. Furthermore, significantly higher hsa‐miR‐762 expression was observed in NPSLE compared with SLE, which inspired us whether much higher hsa‐miR‐762 expression contributed to the development from SLE to NPSLE. Then, the over expression of miR‐762 evidenced that remarkably inhibited the GIPC3 expression. Thus, we suspected that miR‐762 contributed to the development from SLE to NPSLE via suppressing GIPC3 expression. GIPC is a Post‐synaptic density‐95, disks‐large and zonula occludens‐1‐domain containing adapter protein family regulating cell surface expression and endocytic trafficking of multiple components,^28^ and GIPC3 is a member of GIPC. Aberrant expression of GIPC3 has been evidenced to correlate to the audiogenic seizures and sensorineural hearing loss in mouse and human.^29^ Moreover, in Chuvash population, genetic variant of GIPC3 was indicated to associate with hereditary nonsyndromic sensorineural hearing loss.^30^ It follows that the potential correlation between GIPC3 and sensorineural dysfunction deserves further investigation in NPSLE in future.

Although we have revealed the pathogenic role of miR‐762 in SLE and NPSLE for the first time, some limitations were still existed in our study. Owing to few public mRNA data of SLE and NPSLE patients, GIPC3 related bioinformatics analysis was not conducted. Moreover, regarding miR‐762's influence on GIPC3, limited cell lines were employed, and there was a lack of further validation in SLE and NPSLE clinical samples. More detailed role of GIPC3 in SLE and NPSLE patients will be investigated integrating the transcriptome sequencing data in our future work.

## 5 CONCLUSIONS

In summary, via miRNA sequencing, miRNA‐mRNA regulatory network construction, and target gene validation, the significant pathogenic role of hub miRNA miR‐762 in SLE and NPSLE has been revealed for the first time. The upregulation of miR‐762 suppresses the expression of GIPC3 in both SLE and NPSLE samples, which is probably involved in the development of SLE and NPSLE. Furthermore, higher expression of miR‐762 in NPSLE compared with SLE is deserved to be investigated in near future.

## AUTHOR CONTRIBUTIONS

Jijuan Yang and Chun Li contributed to the study conception and design. Material preparation, data collection and analysis were performed by Shuhong Chi and Hongliang Wei. Jijuan Yang, Chun Li, and Wenqing Du conducted the experiments to validate the results of this study. The first draft of the manuscript was written by Jijuan Yang, Chun Li, Shuhong Chi, Hongliang Wei, and Wenqing Du. Qikuan Hu commented on previous versions of the manuscript. All authors read and approved the final manuscript.

## CONFLICT OF INTEREST

The authors declared no conflict of interest.

## ETHICS STATEMENT

This study was performed in line with the principles of the Declaration of Helsinki. Approval was granted by the Scientific Research Ethics Committee of General Hospital of Ningxia Medical University [No. 2019‐487]. Informed consent was obtained from all individual participants included in the study.

## Supporting information

Supporting information.

Supporting information.

Supporting information.

Supporting information.

## Data Availability

The data that support the findings of this study are available from the corresponding author upon reasonable request.
