# Supplementary figures and images for: Upregulation of microRNA‐762 suppresses the expression of GIPC3 in systemic lupus erythematosus and neuropsychiatric systemic lupus erythematosus
Source: Immun Inflamm Dis. 2022 Oct 11;10(11):e719. doi: 10.1002/iid3.719 (PMC9552983; doi:10.1002/iid3.719)

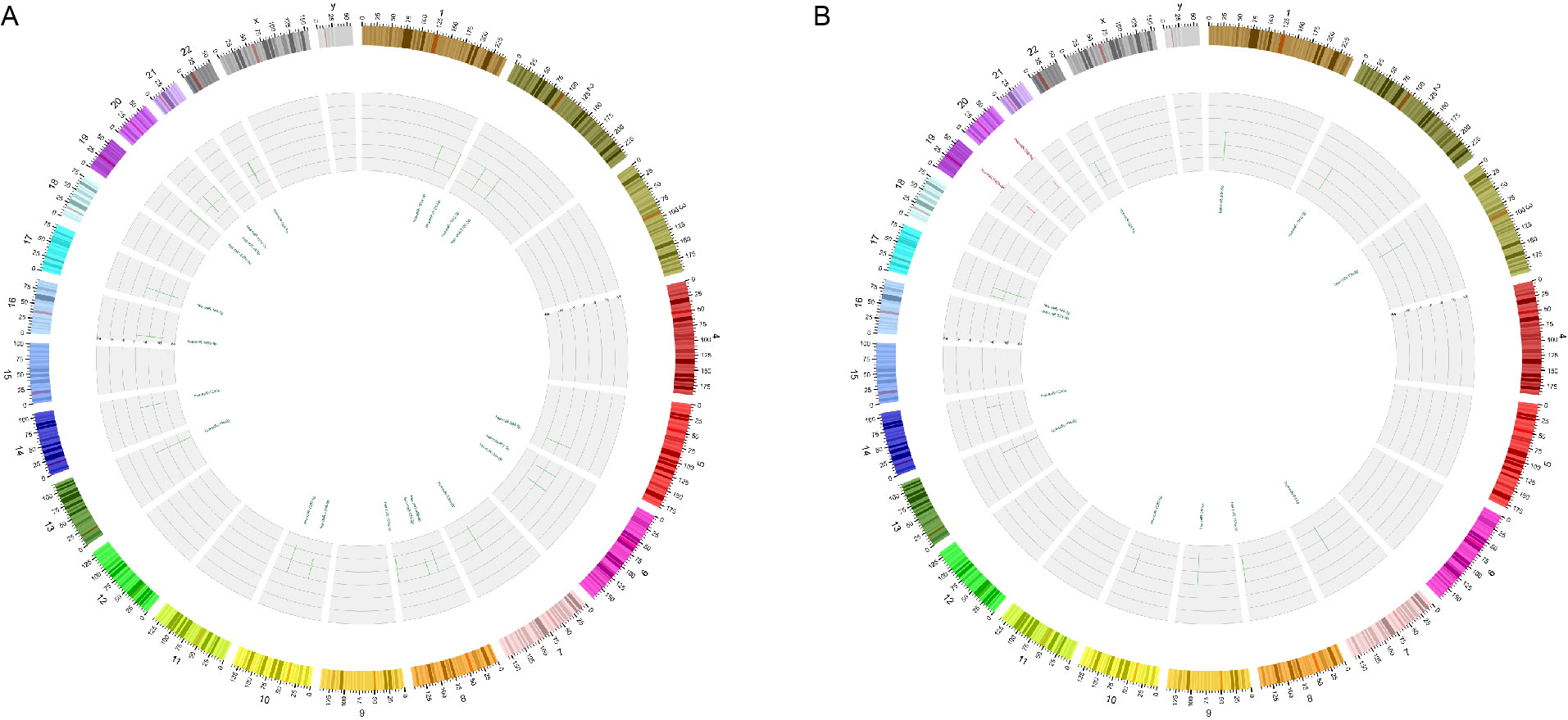

Supplement: Supplementary file 1 — Supporting information. [file IID3-10-e719-s002.jpg]

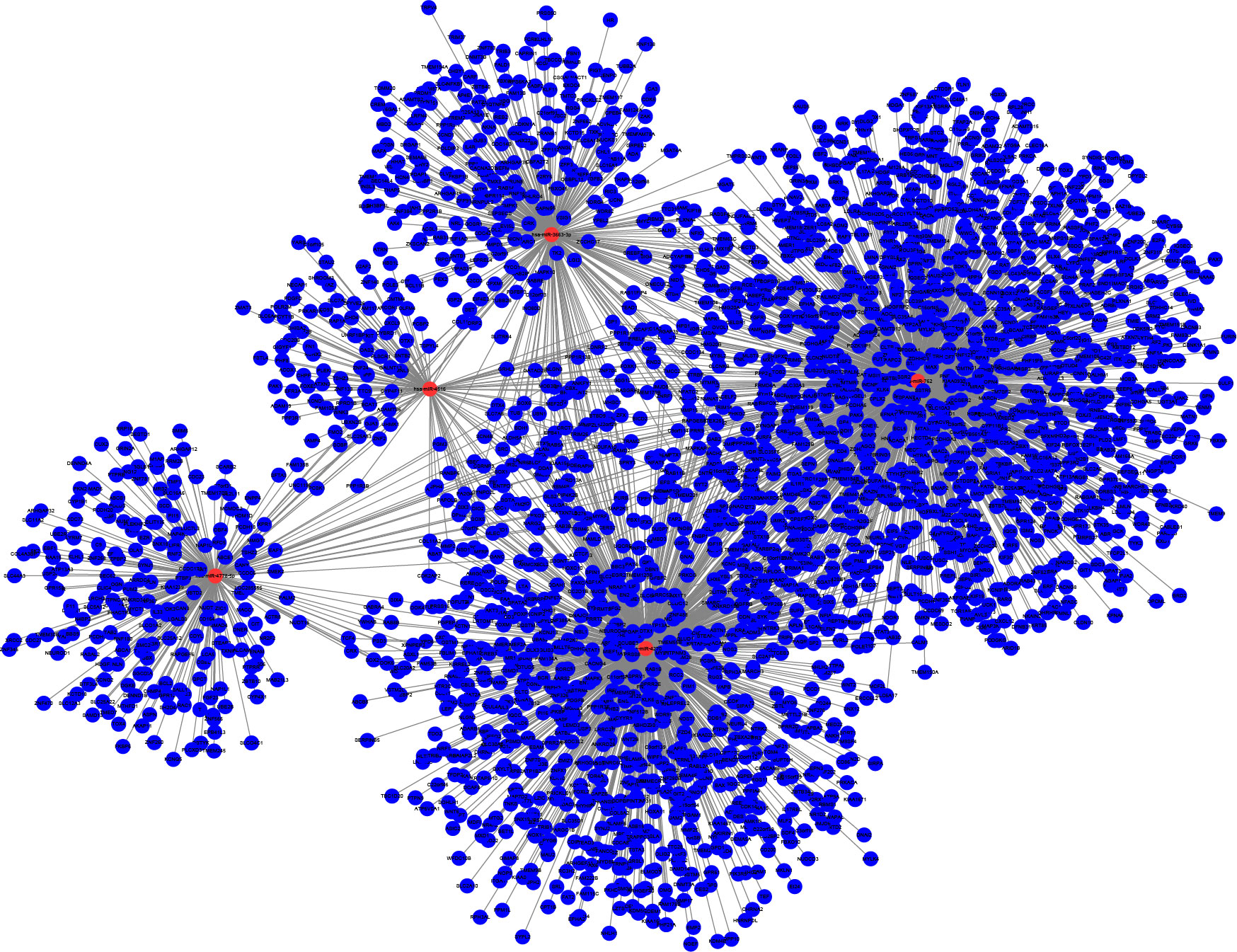

Supplement: Supplementary file 2 — Supporting information. [file IID3-10-e719-s001.jpg]
