# Supplementary material for: Upregulation of microRNA‐762 suppresses the expression of GIPC3 in systemic lupus erythematosus and neuropsychiatric systemic lupus erythematosus
Source: Immun Inflamm Dis. 2022 Oct 11;10(11):e719. doi: 10.1002/iid3.719 (PMC9552983; doi:10.1002/iid3.719)
Supplement: Supplementary file 3 — Supporting information. [file IID3-10-e719-s004.doc]

Table S1. Detailed clinical information of all participants.

|  | No. | Age (years) | Gender | Clinical symptoms | Medical regimens |
| --- | --- | --- | --- | --- | --- |
| NPSLE | 1 | 18 | Female | Repeated facial rash, joint swelling and pain for 10 months, disturbance of consciousness for 6 hours. | 250mg QD of MSS pulse treatment for 3 days, then 80mg QD of MSS intravenous drip, and 0.4g cyclophosphamide injection weekly. |
|  | 2 | 32 | Female | Oral ulcers for half a year, right limb weakness for 3 days. | 500mg QD of MSS pulse treatment for 5 days, then 80mg QD of MSS intravenous drip, and 0.4g cyclophosphamide intravenous drip weekly. |
|  | 3 | 25 | Male | Repeated facial rash for more than 2 years, intermittent seizures for 2 days. | 500mg QD of MSS pulse treatment for 3 days, then 80mg QD of MSS intravenous drip, and 0.4g cyclophosphamide intravenous drip weekly. |
|  | 4 | 22 | Male | Facial and bilateral lower extremity edema for 3 years, headache for 1 month. | 80mg QD of MSS intravenous drip, and mycophenolate mofetil 0.75g BID. |
| SLE | 1 | 23 | Female | Repeated facial rash for 4years. | Prednisone Acetate 30mg QD, Hydroxychloroquine Sulfate 200mg BID, Leflunomide Tablets 20mg QD. |
|  | 2 | 30 | Female | Joint swelling and pain for 3 months, and fever for 1 week. | 40mg QD of MSS intravenous drip, Leflunomide Tablets 20mg QD. |
|  | 3 | 20 | Male | Rash accompanied by puffiness for 1 month. | 40mg QD of MSS intravenous drip, and mycophenolate mofetil 0.5g BID. |
|  | 4 | 26 | Male | Repeated joint swelling and pain for 4 years, rash for 1 month, high fever for 4 days. | 40mg QD of MSS intravenous drip, Hydroxychloroquine Sulfate 200mg BID, Leflunomide Tablets 20mg QD. |
| Controls | 1 | 22 | Female | - | - |
|  | 2 | 20 | Female | - | - |
|  | 3 | 25 | Male | - | - |
|  | 4 | 24 | Male | - | - |

NPSLE: Neuropsychiatric systemic lupus erythematosus; SLE: systemic lupus erythematosus; QD: once a day; BID: twice a day; MSS: Methylprednisolone sodium succinate.
